# Supplementary material for: Diagnostic Accuracy of Dehydroepiandrosterone Sulfate and Corticotropin in Autonomous Cortisol Secretion
Source: Biomedicines. 2021 Jun 28;9(7):741. doi: 10.3390/biomedicines9070741 (PMC8301396; doi:10.3390/biomedicines9070741)
Supplement: Supplementary file 1 [file biomedicines-09-00741-s001.zip › biomedicines-1226207-supplementary.pdf]

Supplemental Table S1. Reference values for DHEA-S adjusted by age and sex*		
**Age (Years)**	**Female DHEA-S (mcg/dL)**	**Male DHEA-S (mcg/dL)**
**18-30**	**83-377**	**105-728**
**31-40**	**45-295**	**57-522**
**41-50**	**27-240**	**34-395**
**51-60**	**16-195**	**20-299**
**61-70**	**9.7-159**	**12-227**
**≥71**	**5.3-124**	**6.6-162**

* Mayo Clinic Laboratories. Dehydroepiandrosterone Sulfate, Serum
https://www.mayocliniclabs.com/test-catalog/Clinical+and+Interpretive/113595

Supplemental Table S2. Diagnostic accuracy parameters for serum DHEA-S and ACTH concentration cutoffs in diagnosing mild autonomous cortisol secretion (MACS)

Cutoff	Sensitivity, % (95% CI)	Specificity, % (95% CI)	PPV, % (95% CI)	NPV, % (95% CI)	False Positive Rate, %
**DHEA-S <40 mcg/dL**	55.0 (47.2 - 62.7)	83.5 (77.8 - 88.2)	72.7 (65.6 - 78.7)	70.0 (66.1 - 73.6)	16.5
**ACTH <10 pg/mL**	53.7 (44.9 - 62.4)	75.3 (67.7 - 81.9)	65.5 (58.0 - 72.2)	65.2 (60.4 - 69.6)	24.7
**DHEA-S < 40 mcg/dL and ACTH <10 pg/mL**	32.1 (24.3 - 40.7)	91.6 (86.0 - 95.4)	76.8 (65.0 - 85.5)	60.8 (57.7 - 63.7)	8.4

ACTH, corticotropin; DHEA-S, dehydroepiandrosterone sulfate; DST, dexamethasone suppression test
MACS was defined as post-DST cortisol of 1.9-5 mcg/dL

Supplemental Table S3. Diagnostic accuracy parameters for serum DHEA-S and ACTH concentration cutoffs in diagnosing nonfunctioning adrenal tumor (NFAT)					
**Cutoff**	**Sensitivity, % (95% CI)**	**Specificity, % (95% CI)**	**PPV, % (95% CI)**	**NPV, % (95% CI)**	**False Positive Rate, %**
**DHEA-S and DHEA-S ratio**					
**DHEA-S >50 mcg/dL**	76.4 (70.1 - 82.0)	65.6 (59.5 - 71.4)	64.8 (60.5 - 68.9)	77.1 (72.2 - 81.3)	34.4
**DHEA-S >80 mcg/dL**	53.8 (46.8 - 60.6)	83.2 (78.1 - 87.6)	72.6 (66.3 - 78.2)	68.5 (65.1 - 71.7)	16.8
**DHEA-S >100 mcg/dL**	42.9 (36.2 - 49.9)	88.7 (84.1 - 92.3)	75.8 (68.3 - 82.1)	65.2 (62.4 - 68.0)	11.3
**ACTH**					
**ACTH >20 pg/mL**	27.3 (20.4 - 35.0)	87.6 (82.4 - 91.6)	60.9 (50.1 - 70.7)	62.9 (60.3 - 65.4)	12.4
**ACTH >25 pg/mL**	14.9 (9.7 - 21.6)	90.8 (86.1 - 94.3)	53.5 (39.6 - 66.9)	60.1 (58.2 - 61.9)	9.2
**ACTH >30 pg/mL**	9.1 (5.1 - 14.8)	94.5 (90.5 - 97.1)	53.9 (35.7 - 71.0)	59.4 (58.0 - 60.8)	5.5
**DHEA-S and ACTH**					
**DHEA-S > 50 mcg/dL and ACTH >20 pg/mL**	24.0 (17.5 - 31.6)	92.2 (87.8 - 95.4)	68.5 (56.0 - 78.8)	63.1 (60.8 - 65.3)	7.8
**DHEA-S > 50 mcg/dL and ACTH >25 pg/mL**	13.0 (8.1 - 19.3)	94.0 (90.0 - 96.8)	60.6 (44.1 - 75.0)	60.4 (58.7 - 62.0)	6.0
**DHEA-S > 50 mcg/dL and ACTH >30 pg/mL**	7.8 (4.1 - 13.2)	96.8 (93.5 - 98.7)	63.2 (40.9 - 81.0)	59.7 (58.4 - 60.9)	3.2
**DHEA-S > 80 mcg/dL and ACTH >20 pg/mL**	17.5 (11.9 - 24.5)	95.4 (91.7 - 97.8)	72.8 (57.4 - 84.4)	62.0 (60.1 - 63.8)	4.6
**DHEA-S > 80 mcg/dL and ACTH >25 pg/mL**	10.4 (6.1 - 16.3)	96.3 (92.9 - 98.4)	66.7 (46.8 - 82.0)	60.2 (58.8 - 61.7)	3.7
**DHEA-S > 80 mcg/dL and ACTH >30 pg/mL**	6.5 (3.2 - 11.6)	97.7 (94.7 - 99.3)	66.7 (41.1 - 85.2)	59.6 (58.4 - 60.7)	2.4
**DHEA-S > 100 mcg/dL and ACTH >15 pg/mL**	24.0 (17.5 - 31.6)	95.9 (92.3 - 98.1)	80.4 (67.2 - 89.2)	64.0 (61.8 - 66.1)	4.1
**DHEA-S > 100 mcg/dL and ACTH >20 pg/mL**	13.0 (8.1 - 19.3)	96.3 (92.9 - 98.4)	71.4 (53.1 - 84.7)	60.9 (59.3 - 62.5)	3.7
**DHEA-S > 100 mcg/dL and ACTH >25 pg/mL**	6.5 (3.2 - 11.6)	97.2 (94.1 - 99.0)	62.5 (38.2 - 81.8)	59.4 (58.3 - 60.6)	2.8
**DHEA-S > 100 mcg/dL and ACTH >30 pg/mL**	4.6 (1.9 - 9.1)	98.6 (96.0 - 99.7)	70.0 (38.0 - 89.9)	59.3 (58.4 - 60.2)	1.4
